# From Pandemic Response to Kill the Clipboard: Patient-Controlled Sharing of Health Data Using International Patient Summary (IPS) and QR codes

**DOI:** 10.1055/a-2902-8515

**Published:** 2026-07-10

**Authors:** John D. D'Amore, Estar Rani Vallapu, Lipika Samal, Bryant T. Karras, William B. Lober

**Affiliations:** 1 Department of Computer Sciences 122593 Boston University Metropolitan CollegeBostonMassachusettsUnited States; 2 More Informatics, Inc.WellesleyMassachusettsUnited States; 3 The Perio Studio Associates PCBostonMassachusettsUnited States; 4 Division of General Internal MedicineDepartment of MedicineBrigham and Women's HospitalBostonMassachusettsUnited States; 5 1811 Department of Medicine, Harvard Medical SchoolBostonMassachusettsUnited States; 6 Department of Health 11153 Washington StateTumwaterWashingtonUnited States; 7 Department of Health Systems and Population Health 49462 University of Washington School of Public HealthSeattleWashingtonUnited States; 8 Department of Health Informatics and Global Health 7284 University of WashingtonSeattleWashingtonUnited States

**Keywords:** standards, HL7, Fast Healthcare Interoperability Resources, continuity of care, patients, 21st Century Cures Act, internet and web technology, consumer health, patient portal, personal health records

## Abstract

**Background:**

Despite advances in interoperability, patient-supplied medical histories commonly use paper-based forms. Dissatisfaction with this inefficiency has led to the “Kill the Clipboard” initiative from the Centers for Medicare & Medicaid Services to advance digital alternatives. During the coronavirus disease 2019 (COVID-19) pandemic, patient-controlled vaccination record sharing demonstrated that Quick Response (QR) codes and digital data standards provide an effective option. Building on this, several nations are using three standards to securely view and exchange medical data: Fast Healthcare Interoperability Resources, the International Patient Summary, and SMART Health Links.

**Objectives:**

This case report explores the use of QR codes and medical data standards in patient-mediated workflows to share medical histories, describing implementation experiences from four such implementations.

**Methods:**

We describe standards and developer community experience with global initiatives and implementations across the United States, Canada, and the Hajj pilgrimage, which all support the use of QR codes in patient-mediated data exchange.

**Results:**

We report on technical readiness, patient adoption, and reported improvements to emergency preparedness using QR codes to share patient summaries.

**Conclusion:**

Advancing patient-mediated exchange with these technologies aligns with initiatives to “Kill the Clipboard.” Preliminary evidence suggests this novel approach can better inform care. These four case studies demonstrate the viability of this approach and the lessons learned from this digital transition.

## Background and Significance

During the coronavirus disease 2019 (COVID-19) pandemic, many state and local governments required individuals to provide proof of vaccination for activities such as indoor dining, public events, and travel. Naturally, individuals found it inconvenient to carry paper credentials, like the Centers for Disease Control and Prevention (CDC) card, which were prone to loss or damage. Several U.S. states, as well as parts of Canada, Singapore, Japan, and Australia adopted digital vaccine cards. 1 These cards allowed individuals to download their health data using the Fast Healthcare Interoperability Resources (FHIR) standard, packaged as a Quick Response (QR) code, to electively share it. 2 3 This approach worked since QR codes were familiar to many, and venues found it easier to scan phones than to inspect paper to comply with government mandates. As the pandemic receded, however, this novel approach to medical data exchange quickly retreated from common use.

### Study Aims and Rationale

This case study investigates the expanded application of QR codes to medical data sharing and describes early implementation experiences on patient-mediated exchange. While most patients today still fill out medical histories on paper, lessons from the COVID-19 pandemic and global case studies can inform future digital health approaches. Digital transition aligns with U.S. federal goals like the “Kill the Clipboard” initiative announced in 2025, which is a voluntary, industry-wide commitment led by the Centers for Medicare & Medicaid Services (CMS) to eliminate repetitive, manual collection of patient information. 4

We begin with a review of current practices in information exchange and standards that enable patient-mediated exchange. We then present patient-mediated digital workflows that have been piloted in several countries using QR codes and the FHIR International Patient Summary (IPS) to inform care transitions.

### Current Standard of Care in Information Exchange

**Fig. 1:**
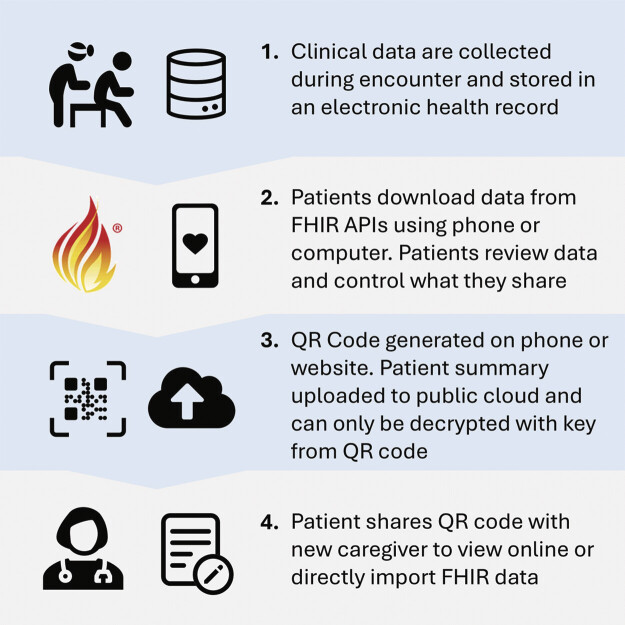
Patient-mediated workflow for data sharing using FHIR and QR codes. API, application programming interface; FHIR, Fast Healthcare Interoperability Resources; QR, Quick Response.

The current standard of care for collecting a patient's health history is a written form filled out by the patient, followed by a discussion with a clinician. 5 6 This information is typically verbally confirmed and recorded with clinical judgment into the medical record. Exchange of medical histories through Epic's Care Everywhere, DirectTrust, networks operating under the Trusted Exchange Framework and Common Agreement (TEFCA), or other health information exchanges complements but does not replace this activity. 7 Network-facilitated medical histories provide information directly from a known source without modification, but these records are generally not reviewed by patients, are difficult to import, and may contain out-of-date or contradictory information. 8 9 Although patients provide the most recent details about their health, their accounts may be incomplete. Research has found that patients commonly miss key information, particularly among those with many comorbidities or high polypharmacy. 10 11 Even when patients arrive with comprehensive records, many organizations have policies to disallow media use and force manual data re-entry. 12 Prior attempts to transition from paper-based approaches showed that digital records must be reviewed and controlled by the patient, be easily accessible to clinical systems, and adhere to widely accepted data standards. 13 14 Previous personal health record initiatives, such as BlueButton and Apple HealthKit, lacked a common method to present this information to electronic health records (EHRs). 15 Since internet usage and FHIR application programming interfaces (APIs) are now prevalent in U.S. health care settings, a patient-mediated digital workflow as proposed in Fig. 1 provides an opportunity for such a transition.

### SMART Health Links to Securely Share Data

While internet use is commonplace, there is no predominant way to securely access health data through uniform resource locators (URLs). This challenge has been addressed through the development of a standard called SMART Health Links (SHLs). An extension of SMART Health Cards, originally developed in 2021 in conjunction with digital vaccine cards during the COVID-19 pandemic, the SHL standard is published by Health Level Seven International (HL7). 16 SHLs leverage web signatures and cryptography to provide a secure approach to medical data sharing across the internet. The resulting URLs can be packaged into QR codes compatible with mobile devices and recognizable by the lay public.

**Fig. 2:**
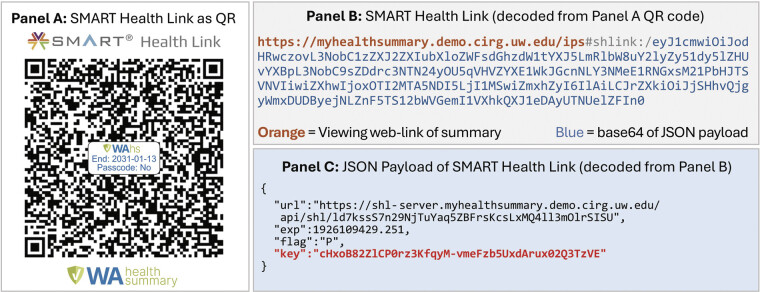
The QR code in Panel ( **A** ) contains an SHL which can be read by a phone. SHLs are primarily designed to share FHIR data, and an illustrative patient summary will display when the link is opened. A user creating an SHL can also directly copy the URL from the QR code, which is shown in Panel **(B)** . The URL fragment after “shlink” contains a base64-encoded JSON payload that contains the following, as shown in Panel ( **C** ): the server location to retrieve the summary, an optional expiration time of the SHL, a flag if data may be protected by a passcode, and a 256-bit key shown in red as needed to decrypt the data. The fictional data accessed by the QR code was downloaded from the Inferno on HealthIT.gov suite and extended by personnel working with Washington Health Summary. 64 FHIR, Fast Healthcare Interoperability Resources; SHL, SMART Health Link; QR, Quick Response; URL, uniform resource locator.

People have grown familiar with QR codes in the past decade, with surveys showing over 80% adoption. 17 18 QR codes are commonly used by retail stores for discounts, restaurants for digital menus, and airlines for boarding information. While medical data packaged inside QR codes were sufficient for digital vaccine cards, the amount of data is limited by the physical size and practical resolution of the QR image. A single QR code can hold a few kilobytes of medical data, reasonable for a few vaccines, but insufficient for a full medical history. SHLs, in contrast, do not embed the medical information inside the QR codes. Instead, the QR code embeds a URL with two parts: The first provides a reference web link to view the clinical information, and the second part encodes a URL fragment, providing the download location and cryptographic key to the data as detailed in Fig. 2 . While fundamentally just a URL, SHLs contain 256 bits of entropy, like cryptocurrencies and other encryption technologies, making data inaccessible to users without the key. SHLs also allow additional protections such as a passcode and link expiration dates, both of which are implemented at the web link, typically an internet server, for added security.

SHLs are designed to have FHIR resources as the primary information payload. The specific FHIR information in an SHL is flexible by design and is being evaluated for health insurance cards and multiple other applications. 19 For the use case of SHL to provide a medical history, the FHIR IPS is a standards-based container for that information. 20

### International Patient Summary

The IPS is a medical document summary composed of 16 sections, many of which correspond to information commonly collected using clipboards today. Most prominent are the mandatory sections on patient allergies, medications, and problems. By design, the IPS is flexible and may include any data that support its use in cross-border care. The IPS recommends the use of the Systematized Nomenclature of Medicine (SNOMED) as a primary terminology for encoding medical information for global interoperability. 21 While not all countries use SNOMED for regional or national encoding, it represents a global standard where worldwide code usage is permitted. 22 HL7 originally published the FHIR IPS in 2020 and has since incorporated significant feedback from implementers in its second version, published in 2025. 20

**Fig. 3:**
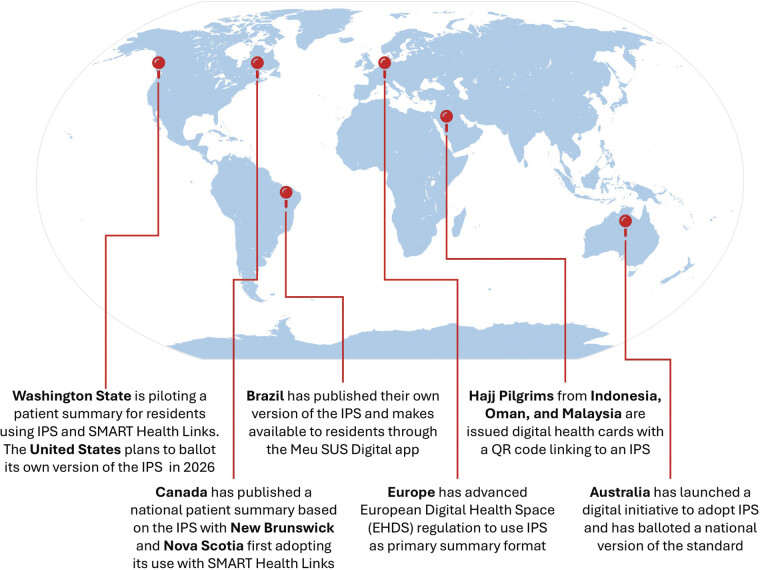
Select regions implementing the IPS for care summary exchange. IPS, International Patient Summary.

The IPS is a global standard recognized by the International Standards Organization (ISO), the European Committee for Standardization (CEN), HL7, and Integrating the Healthcare Enterprise (IHE), and is already adopted by multiple governments and the G7. 23 24 25 Major EHRs, such as Epic, now support its use to share information during care transitions. 26 The cross-border use case of the IPS includes travel across national borders, but many nations are adopting it as a domestic basis of exchange as well. Canada, Brazil, Australia, and the European Union have selected the IPS as the primary domestic basis for care summary exchange, and many regions are actively advancing implementation, as shown in Fig. 3 . 27 28 29 30 One international initiative with over 40 member countries is the Global Digital Health Partnership (GDHP). The GDHP interoperability workstream has made patient-mediated IPS exchange a priority, which aligns with the workflow reviewed in this article. 31

### Case Study Selection Methods

This study employed a purposive case study selection approach to identify representative implementations of the IPS with QR codes. Case studies were selected based on the authors' direct involvement in IPS-related development activities, such as connectathon participation, and personal communication with ongoing implementations across vendors and geographies through the GDHP. Candidate initiatives were included if they (1) demonstrated a concrete implementation aligned with the IPS and QR codes and (2) represented distinct organizational, geographic, or technical implementation contexts. The intent of this selection approach was not to provide an exhaustive survey, but rather to illustrate different implementation patterns observed by the authors through multiyear participation in the IPS ecosystem.

Four implementations were selected from Washington State, Canada, the Hajj pilgrimage, and vendor apps to demonstrate the viability of pairing the IPS with QR codes during care transitions.

### Washington State Evolution from COVID Vaccines to Health Summaries

The Washington Department of Health maintains the state's immunization registry and pioneered digital vaccine cards during the COVID-19 pandemic through the WA Verify initiative, available at https://waverify.doh.wa.gov/ . WA Verify supports access in over 52 languages for Washington residents and has been accessed nearly 2 million times since its 2021 launch. 1 2 3 32 33 To support patient-centered care, engagement beyond the pandemic, and facilitate patient management of complex information, there was a desire to broaden the scope of information sharing beyond digital vaccine cards. Data from a WA Verify postusage assessment indicated a strong interest among WA Verify users in patient-controlled record sharing, as well as a willingness to engage through an app, including public health advisories and initiatives. 34

In 2023, the Department of Health launched an initiative known as Washington Health Summary (WAHS) to add medical information like problems, allergies, medications, and advance directives. 35 36 WAHS selected the FHIR IPS as the primary format for data sharing and draws data from mandated FHIR APIs in both provider and insurer settings. WAHS also uses FHIR Implementation Guides for patient-contributed information such as advance directives and occupational history. 37 38 Where needed, WAHS implements FHIR facades and translation layers to convert legacy sources such as state immunization or prescription data to FHIR. WAHS creates SHLs both as QR codes and links for patient-facilitated health summary exchange, as shown by the illustrative QR code in Fig. 2 .

While the United States has over a decade of health information exchange using HL7's Clinical Document Architecture (CDA), this format is less accessible to patients and does not leverage the newer FHIR standards cited in federal regulations. The work in Washington State demonstrates the feasibility of federal government efforts to develop a U.S. patient summary based on the FHIR IPS. 39 40 In addition, while Washington benefits from an emerging state-wide authentication initiative for government services, WAHS is designed to be reproducible by other organizations as a social good, through the use of modern web technologies and data standards, containerized server deployment, modular authorization/authentication, and use of SMART on FHIR protocols. While this patient-centric data exchange model is early in U.S. implementation, it complements provider-mediated exchange and provides an opportunity to supplement care summaries with additional data. These include patient-supplied information and advance care planning documents, such as Portable Orders for Life Sustaining Treatment (POLST) and Do Not Resuscitate (DNR) information, as well as information from payers and public health. It also provides patients with an elective mechanism to engage directly with public health around topics such as health monitoring and personalized decision support.

## Canadian International Patient Summary Rollout in New Brunswick and Nova Scotia

Canada's health system has long relied on paper-based data exchange. Providers commonly use fax or portable document format (PDF) to share patient information as patients move within or between provinces. Unlike the United States, Canada did not adopt HL7's CDA as a national standard for structured data to be shared with downstream systems. 28 Consequently, paper-based formats lead to variability in the format and type of information exchange, which in turn burdens receivers. 41 In response, Canada Health Infoway, the nation's health authority for information exchange, began adopting a standards-based patient summary in 2021. They published a Pan-Canadian Patient Summary (PS-CA) for provider-to-provider exchange and patient-mediated exchange based on the FHIR IPS. Individual provinces have adapted this national standard to regional implementations, and the PS-CA now provides both a reference implementation and a framework for harmonization. 42 43

A patient-mediated approach using PS-CA launched first with the capability to share a summary with anyone in their circle of care beyond just typical care providers. 44 In 2024, New Brunswick launched the MyHealthNB app, where all residents can generate an SHL that points to an IPS-conformant summary containing allergies, medications, problems, immunizations, and laboratory results. This allows patients to “cross both provincial and international borders equipped with a shareable, digital set of basic clinical data.” 45 While usage is expected to grow over time, over 2,000 New Brunswick residents have already accessed their summaries, with 43% sharing with their care provider and 20% sharing with other family members or caregivers. 46 Preliminary findings from a province-wide patient survey of 107 New Brunswick patient summary users report significantly greater emergency preparedness ( *p* = 0.021), improved ability to avoid care duplication ( *p* < 0.001), decreases in emergency department utilization ( *p* < 0.001), and higher engagement in healthy behaviors ( *p* = 0.022), when compared with 778 non-users. 47 While these survey results are from a small cross-sectional sample and subject to response and selection biases, they provide early quantification of patient perspectives on summary use.

The New Brunswick launch motivated neighboring Nova Scotia to launch a similar initiative in 2025. As Matthew Clarke, Associate Chief Medical Information Officer at Nova Scotia Health, states, “We often see patients from across health provincial health jurisdictions with limited to no access to patient data. As an Emergency Department physician with increasing patient complexity, the more I know about my patients, the better I can manage and support their care. I had an experience where I was practicing in a rural emergency department in Nova Scotia and saw a patient from New Brunswick. They were able to share with me an accurate medication list, as well as investigations they'd had to date, which led to a meaningful change in next steps, including avoiding delays and risks associated with repeat investigations. In Nova Scotia, we've been able to go a step further and build a secure data environment that includes patient information from across community, primary care, and hospital records. As a result, we have an incredibly comprehensive patient summary, which patients can now share with whomever they choose, putting them in control of their information.” 48 49 Canadian technology vendors have demonstrated similar approaches for other provinces, which are expected to follow the lead of New Brunswick and Nova Scotia in the coming months.

## International Patient Summary for the Hajj Pilgrimage

The Hajj pilgrimage is one of the world's largest annual mass gatherings, with pilgrims from more than 180 countries. 50 In 2024, Saudi Arabia hosted 700,000 international and 140,000 domestic pilgrims. Tragically, more than 1,300 died that year, largely among pilgrims exposed to extreme heat. 51 Common health risks documented in this unfortunate outcome included respiratory infections, cardiovascular events, and other comorbidities, especially among elderly pilgrims. 52

To better communicate health information during the pilgrimage, the World Health Organization (WHO) has promoted digital health innovation and leveraged investments from the COVID-19 pandemic. In 2023, the WHO adopted the European Union's Digital COVID Certificate framework and scaled it into the Global Digital Health Certification Network (GDHCN), extending its trusted verification model beyond COVID. Through the GDHCN, WHO supports mobile-based QR codes to communicate vaccination records, allergies, and medical histories using an IPS. These tools can enhance emergency response and cross-border care information exchange during the Hajj. 53

Beginning in 2024, the countries of Oman, Indonesia, Malaysia, and Saudi Arabia adopted this initiative. Like New Brunswick and Nova Scotia, these countries relied on patients to carry a QR code to access their medical information. The QR code could be on a mobile device or a physical card associated with travel visas. In contrast to the United States and Canada, the GDHCN adopted a more constrained access modality where only trusted providers on the network can access data due to privacy and audit requirements. To technologically limit access, the WHO developed the Verified Health Link standard, similar to SHL, and work has started within the HL7 and WHO communities to align these approaches. 54 In total, over 200,000 pilgrims have been able to access their IPS through the GDHCN. Over 80 WHO member states have adopted GDHCN standards, and usage for more nations and beyond the Hajj will be possible in the coming years. 55 56

## CommonHealth and Patient-Centered Apps

The Commons Project is a U.S. non-profit organization that allows individuals to gather their FHIR data through interfaces mandated by the 21st Century Cures Act. Their free app, called CommonHealth, is available on Android devices and connects to over 12,000 data sources. 57 After gathering information on a patient's phone, CommonHealth allows patients to package individual data into an IPS. Then, an SHL is created on the patient's phone with the encrypted IPS payload uploaded to a secure cloud to facilitate data sharing. 58

While CommonHealth is primarily focused on U.S. consumers, many nations promote patient rights to access and share medical data. Similar apps may be developed by for-profit, non-profit, or governmental organizations. For example, Brazil's Ministry of Health now supports IPS creation and sharing through the Meu SUS digital health app. 27 The growth of technologies that allow patients to access, manage, and share their health information turns long-standing patient empowerment and advocacy goals into reality. 59 60 61

## Discussion

“Kill the Clipboard” is about replacing redundant paper intake forms with digital tools that fit into clinical workflows. These tools must address two barriers: A simple mechanism to present data and a common payload format. For purposes of informing digital care transitions, pairing SHLs with the FHIR IPS is a logical starting place. This patient-mediated workflow allows sharing structured health data via secure mechanisms without needing paper or multiple portal logins. The dual nature of SHL also allows gradual onboarding for implementers. Scanning an SHL with IPS provides a viewable web site to assist with filling out intake forms today, and then as FHIR adoption matures, the same QR code can be used to programmatically import the data into EHRs.

### Workflow and Content Limitations

While the case studies in this article demonstrate progress since the pandemic across multiple countries, advancing this approach, workflow and content limitations remain. While a patient-mediated workflow helps streamline information during intake, data still require reconfirmation by clinicians and reconciliation with information from verified health institutions. This limits the time savings associated with this digital transition. In addition, while the IPS standard provides a baseline of data expectations, different organizations may generate summaries with varying terminologies and content. While SNOMED makes its core content freely available worldwide, its use is not required in the standard, and translation from national vocabularies to SNOMED is not universal. 20 21 22 For example, many nations record immunization information in other vocabularies (i.e., CVX in the United States), and while initiatives are underway to facilitate SNOMED mapping, they are still incomplete. Some IPS sections also go beyond intake information, which may result in an additional clinical burden to review.

### Balancing Access with Security

The open SHL approach to sharing information also limits access control and audit capabilities, potentially impacting security and privacy. In its simplest form, only the key from the QR code is needed to decrypt and access stored information. Since users do not log into a network, access can be anonymous and possibly malicious. While SHL implementations may include passcodes, link expiration dates, and revocation, these features are not required of an SHL server. 35 58 Moreover, even when available, there is not yet evidence that users will effectively use them. Sharing an SHL without security constraints is equivalent to handing over a physical medical summary, which may be desirable in unplanned emergencies and precarious when a phone or QR code is lost.

Furthermore, data from SHLs can mix patient- and provider-authored content within an IPS. Tracking the provenance of attested or verified information after data import is available in FHIR but has not been commonly adopted to date. Audit, security, and privacy considerations led the Hajj pilgrimage use case to restrict their QR codes to clinical data from origin-country health facilities and users authorized on the GDHCN, which provides a framework for auditing and use verification. Additional work will be needed to fully harmonize QR codes between SHL and Verified Health Links in the coming years. 16 54

Finally, QR code data sharing is often facilitated using smartphones. While smartphone availability and QR code digital literacy are increasing globally, a significant population remains where access is limited. Implementers of QR code workflows should consider alternatives to the phone display of codes, such as printed QR codes for Hajj pilgrims and Washington State residents, as well as image and link sharing. 1 56

### Advancing Future Implementations

Acknowledging those limitations, the next step is for EHRs and providers to change intake processes. Previous research demonstrates that FHIR data structures can digitize paper processes into usable digital workflows. 62 63 EHR vendors and providers can reference implementation guides and conformance tooling that have been released to align and assist future implementations with these workflows. These include the Inferno Test Kit supporting IPS, implementation guidance from both WHO and HL7, and testing at global and regional connectathon events. 16 20 54 64 65 This will require time and money, but the return on those investments can be more complete records and a patient experience that aligns with other industries. People routinely present QR codes to board a plane and pay for services. Now, a similar digital transition is available for health care. Washington state, Canada, the Hajj pilgrimage, and CommonHealth demonstrate a path for future improvement.

It is important, however, to acknowledge that clinical improvements cannot be realized through technical innovation alone; information reconciliation and communication workflows remain deeply connected to patient safety, regulatory needs and legal requirements. Future research should explore necessary workflow changes and more rigorously quantify the preliminary improvements from these case studies.

## Conclusion

Multiple countries have demonstrated that patients can use the IPS to share health data with SHLs via QR codes. This is a novel method to effect medical data exchange. These initiatives build on initial work from the COVID-19 pandemic and complement FHIR initiatives underway both in the United States and abroad. While evidence of clinical benefit is preliminary and anecdotal, these technologies can reduce the friction of fax communications and paper-based forms and decrease the burden of duplicate data entry. As future initiatives seek to “Kill the Clipboard,” these technologies hold significant potential.

## Notes

**Clinical Relevance Statement**
Patient-mediated data exchange with the use of QR codes and interoperability standards presents a practical alternative to paper-based clinical workflows globally. Case studies in this paper demonstrate the experiences of early implementers and how this approach may improve information exchange during care transitions. In addition, these tools may reduce the administrative burden and provide timely support in clinical decision-making.

## Notes

**Multiple-Choice Questions**

1. When a patient uses a SMART Health Link to share a medical summary, which of the following is true?
  a) The patient summary is within the QR code.
  b) The QR code points to a server where the unencrypted summary resides.
  c) The QR code points to a server where the encrypted summary resides.
  d) The QR code points to a server where providers must authenticate to access the summary.
  **Correct Answer** : The correct answer is option c. A patient summary is often too large to fit inside a QR code. SMART Health Links contain a cryptographic key that encrypts the data, which are then uploaded to a server. SMART Health Links allow anyone with the key and an optional passcode to access the data and do not require users to authenticate before accessing. The desire for user authentication is why the Verified Health Link standard, similar to SMART Health Links, has been developed by the World Health Organization for the Hajj pilgrimage.
2. Which of the following is **not** true of the International Patient Summary?
  a) The IPS is a global standard recognized by the International Standards Organization (ISO), the European Committee for Standardization (CEN), Health Level Seven International (HL7), and Integrating the Healthcare Enterprise (IHE).
  b) The IPS requires the sections on allergies, medications, and problems.
  c) The IPS requires the usage of the Systematized Nomenclature of Medicine (SNOMED).
  d) The IPS is being adopted by many geographies worldwide.
  **Correct Answer** : The correct answer is option c. The IPS recommends the use of SNOMED but does not require it. The mapping of regional vocabularies to SNOMED remains a challenge worldwide, and its required use would limit the potential adoption of IPS.
3. What has New Brunswick's adoption of SMART Health Links to share summaries shown?
  a) Patients may often share their summaries with family or other caregivers.
  b) Patients did not effectively use the technology due to unfamiliarity with QR codes.
  c) The province has seen a 15% reduction in ER visits.
  d) The province plans to discontinue use due to poor adoption.
  **Correct Answer** : The correct answer is option a. 2025 data show that patients often share a patient summary with other family members or caregivers outside of a typical care encounter. Patient summaries using SMART Health Links can empower patients to share their data with whomever they choose.
4. What network has the Hajj pilgrimage begun using to support data sharing?
  a) The International Network for Patient Summaries, managed by the World Health Organization.
  b) The Global Digital Health Certification Network, managed by the World Health Organization.
  c) The Interoperability Workstream of the Global Digital Health Partnership.
  d) EHR Networks, such as Epic's CareEverywhere, to securely share data.
  **Correct Answer** : The correct answer is option b. In 2023, the Global Digital Health Certification Network, managed by the World Health Organization, was launched as an evolution of prior pandemic work on digital vaccine sharing. This network has been used since 2024 to support patient summary exchange as part of the Hajj pilgrimage.
